# Electrokinetic instability in microchannel ferrofluid/water co-flows

**DOI:** 10.1038/srep46510

**Published:** 2017-04-13

**Authors:** Le Song, Liandong Yu, Yilong Zhou, Asher Reginald Antao, Rama Aravind Prabhakaran, Xiangchun Xuan

**Affiliations:** 1 School of Instrument Science and Opto-electronic Engineering, Hefei University of Technology, Hefei 230009, China; 2 Department of Mechanical Engineering, Clemson University, Clemson, SC 29634-0921, USA

## Abstract

Electrokinetic instability refers to unstable electric field-driven disturbance to fluid flows, which can be harnessed to promote mixing for various electrokinetic microfluidic applications. This work presents a combined numerical and experimental study of electrokinetic ferrofluid/water co-flows in microchannels of various depths. Instability waves are observed at the ferrofluid and water interface when the applied DC electric field is beyond a threshold value. They are generated by the electric body force that acts on the free charge induced by the mismatch of ferrofluid and water electric conductivities. A nonlinear depth-averaged numerical model is developed to understand and simulate the interfacial electrokinetic behaviors. It considers the top and bottom channel walls’ stabilizing effects on electrokinetic flow through the depth averaging of three-dimensional transport equations in a second-order asymptotic analysis. This model is found accurate to predict both the observed electrokinetic instability patterns and the measured threshold electric fields for ferrofluids of different concentrations in shallow microchannels.

Electrokinetic flow is the fluid motion generated by an external electric field^1^,^2^. It has a much smaller resistance than the traditional pressure-driven flow^3^, and is the preferred mode for transport of fluids and samples in microfluidic devices^4^,^5^,^6^,^7^. This is attributed to its nearly plug-like velocity profile^8^, which yields a much smaller sample dispersion than the parabolic pressure-driven flow^9^. However, due to the direct dependence on fluid (e.g., viscosity and permittivity) and channel (e.g., zeta potential) properties^10^, electrokinetic flow (or more specifically, electroosmosis^11^) loses its favored plug-flow feature and may even become unstable in some circumstances. For instance, Joule heating, which is the generation of heat in the fluid due to its resistance to electric current^12^, has been demonstrated to induce axial pressure-driven flow in capillary electrophoresis due to the temperature dependence of fluid properties^13^,^14^. It can even cause electrothermal flow^15^ in the form of counter-rotating vortices^16^,^17^,^18^,^19^ due to the action of electric field on the thermally induced fluid property gradients^20^,^21^. Fluid circulations can also be produced in electrokinetic microchannel flow when there is a non-uniform surface property due to, for example, heterogeneous patterning^22^,^23^, field effect^24^,^25^ or induced charge effect^26^,^27^,^28^,^29^,^30^.

Another circumstance involving fluid property variation is the electrokinetic flow of two fluids that are either displacing^31^,^32^ or co-flowing^33^,^34^ with each other in single microchannels. The electrokinetic displacement of fluids takes place in, for example, zeta potential measurement via electric current monitoring^35^ and sample stacking via isotachophoresis^36^ etc. The electrokinetic co-flow of two or more fluids with dissimilar properties is often encountered in microfluidic mixing^37^,^38^. Strong electrokinetic instabilities^39^,^40^,^41^ with chaotic^42^ or even turbulent^43^ flows have been reported to occur at the interface of two electrolytes with different electric conductivities though the Reynolds number remains low. They are attributed to the electric body force that acts on the induced free charge inside the mixing zone^44^,^45^. The mismatch of electric conductivity can also be achieved by adding colloids into one of the co-flowing fluids^46^. A similar idea has recently been demonstrated by our group^47^, which studied the electric field-driven instability in ferrofluid (a suspension of superparamagnetic nanoparticles^48^) and water co-flows through a T-shaped microchannel. This electrokinetic mixing method frees the hydrodynamic pumping that is required in magnetic field-driven mixing of ferrofluid and water^49^,^50^. We have developed a two-dimensional (2D) numerical model to understand the electrokinetic instability in microchannel ferrofluid/water co-flows. While the dynamic behaviors of the interface are qualitatively simulated, the predicted threshold electric fields for sustainable flow instabilities are substantially smaller than the experimental data^47^. Such a significant discrepancy must be due to the limitation of a regular 2D model that ignores the top and bottom channel wall effects.

We develop in this work a depth-averaged numerical model for an improved quantitative simulation of both the interfacial electrokinetic behavior and the threshold electric field for the onset of electrokinetic instability in microchannel ferrofluid/water co-flows. This model accounts for the potential influences of the top and bottom channel walls on charge, fluid and mass transfer in shallow microchannels via the depth averaging of standard transport equations. The validity and accuracy of this model are tested by comparing its predictions with the experimental measurements and as well the predictions of a regular 2D model in channels of different depths and for ferrofluids of different concentrations.

## Results and Discussion

### Effect of electric field

**Figure 1:**
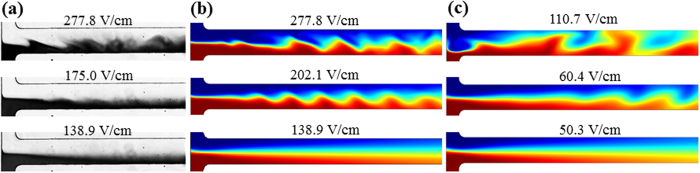
Electric field effect on the interfacial behavior of the ferrofluid (0.2× EMG 408) and water co-flow in a 45 μm deep T-shaped microchannel: (**a**) experimental images (dark for ferrofluid and white for water); (**b**) predicted ferrofluid concentration fields (red for ferrofluid and blue for water) from the depth-averaged numerical model; (**c**) predicted ferrofluid concentration fields from a regular 2D model. The experimental and numerical images are each obtained at 20 s after the corresponding DC electric field (value being labeled on the image) is applied. Note that the regular 2D simulation in (**c**) is each performed at a much lower electric field than the depth-averaged simulation in (**b**) because, otherwise, chaotic waves occur in every case of (**c**).

Figure 1 shows the electrokinetic behaviors of the ferrofluid/water co-flow under three different DC electric fields. The T-shaped microchannel is 45 μm deep and the ferrofluid is 0.2× EMG 408. Under a small electric field like 138.9 V/cm (estimated from a 250 V applied voltage drop across an overall 1.8 cm long channel), no instabilities are observed at the ferrofluid and water interface in Fig. 1(a) (bottom) where only molecular diffusion takes place. This observation is consistent with the predicted ferrofluid concentration field from the depth-averaged model in Fig. 1(b) (bottom). When the electric field is increased to 175.0 V/cm, stable periodic fluid waves can be visually identified at the interface in Fig. 1(a) (middle). Similar to our earlier paper^47^, this value is defined as the experimental threshold electric field. The numerical threshold electric field from the depth-averaged model is 202.1 V/cm in Fig. 1(b) (middle), which is 15.5% higher than the experimental value. This is opposite to the prediction from a regular 2D model where the numerical threshold electric field is only 60.4 V/cm as viewed in Fig. 1(c) (middle). At an electric field that is much higher than the experimental threshold value like 277.8 V/cm (corresponding to a 500 V voltage drop across the channel), chaotic waves are experimentally observed in Fig. 1(a) (top). A similar phenomenon is also predicted by the depth-averaged model at the same electric field in Fig. 1(b) (top). In contrast, the regular 2D model predicts the occurrence of chaotic waves at an electric field (110.7 V/cm) that is even smaller than the experimental threshold electric field; see Fig. 1(c) (top). This indicates the strong stabilizing effects on electrokinetic flow from the top and bottom channel walls. In addition, consistent with the experimental observation, the predicted instability waves from the depth-averaged model in Fig. 1(b) (middle) are inclined towards the upstream of the flow. On the contrary, those from the regular 2D model in Fig. 1(c) (middle) are inclined towards downstream.

**Figure 2:**
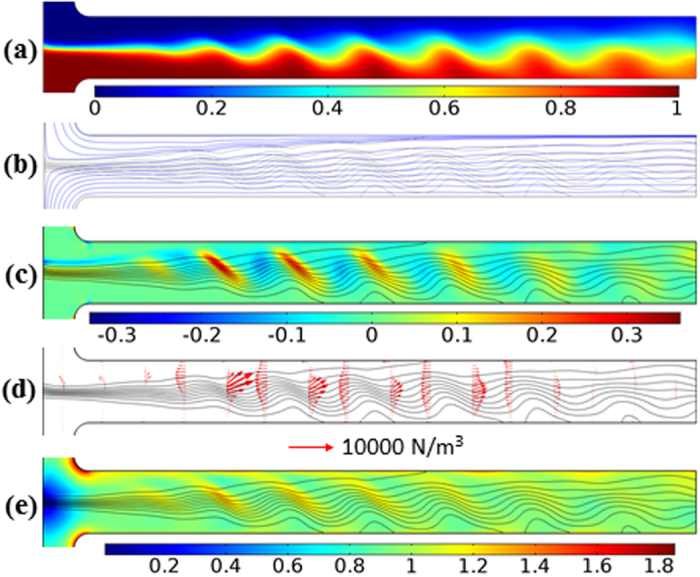
Numerically predicted field distributions from the depth-averaged model for the 0.2× ferrofluid/water co-flow in a 45 μm deep T-shaped microchannel under the threshold electric field: (**a**) ferrofluid concentration (contour), (**b**) electric field (lines), (**c**) free charge density (contour, C/m^3^), *ρ*_*e*_, (**d**) electric body force (vector plot), and (**e**) fluid velocity (contour, mm/s). The background curving lines in (**b**) to (**e**) represent the contour lines of ferrofluid concentration in (**a**).

Figure 2 shows the predictions of other property fields from the depth-averaged model at the numerical threshold electric field in a 45 μm deep microchannel. For a better reference, the plot of concentration field is included in Fig. 2(a). Also, the contour lines of ferrofluid concentration are retained in the other sub-plots of Fig. 2 to relate each field to the instability waves. As viewed from Fig. 2(b), the electric field lines coming from the ferrofluid inlet quickly focus those from the water inlet into a thin layer near the sidewall of the main-branch. This is a consequence of the significant mismatch between ferrofluid and water electric conductivities. The electric field lines in the main-branch are mainly parallel to the sidewalls though slight periodic waves seem to occur in the near T-junction region. The positions of these weak electric field “waves” are consistent with those of the ferrofluid concentration waves due to the concentration dependence of electric conductivity. Figure 2(c) presents the distribution of free charge density, *ρ*_*e*_, which becomes positive in the ferrofluid side and negative in the water side within the diffusion zone that immediately follows the T-junction. This produces an anti-clockwise electric body force, *ρ*_*e*_**E**, which, as viewed from Fig. 2(d), deforms the ferrofluid/water interface yielding instability waves that are convected downstream by electroosmosis. The resulting mixing of the two fluids reduces the magnitude of electric body force such that the fluid waves gradually damp out at the channel downstream. Moreover, the free charge density changes sign from negative at the fore to positive at the rear of every single wave in Fig. 2(c). The electric body force thus stretches the fluid wave leading to an increased wavelength and a decreased amplitude downstream at the channel. It also decelerates and accelerates the flow at the fore and rear of the instability waves causing the periodic variation of fluid velocity in Fig. 2(e).

### Effect of channel depth

**Figure 3:**
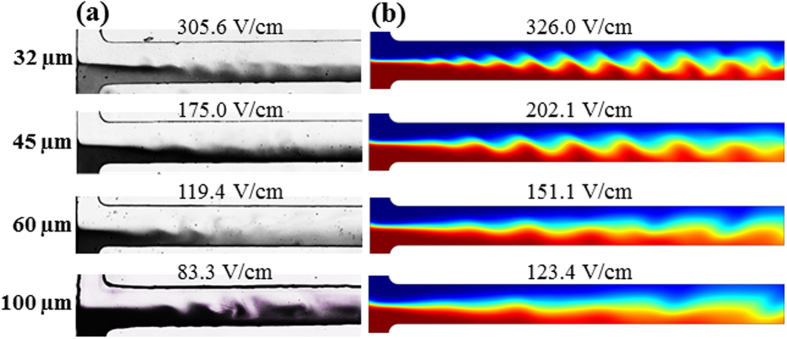
Channel depth effect on the electrokinetic instability of the ferrofluid (0.2× EMG 408) and water co-flow at the threshold electric field: (**a**) experimental images (dark for ferrofluid and white for water); (**b**) numerical predictions of ferrofluid concentration field (red for ferrofluid and blue for water) from the depth-averaged model. All images are each obtained at 20 s after the corresponding experimental or numerical threshold electric field (labeled on the image) is applied.

Figure 3 shows the effect of channel depth on the electrokinetic instability of 0.2× EMG 408 ferrofluid and water co-flow. The experimental images at the threshold electric fields in Fig. 3(a) indicate that the instability waves have an extended wavelength (i.e., less number of waves within a certain channel length) in a deeper microchannel. This is believed to be associated with the decreasing threshold electric field as a result of the reduced top/bottom wall suppression effects on electrokinetic instability. Hence, the instability waves are convected downstream by a smaller electroosmotic flow in a deeper microchannel. Their amplitude does not seem to vary significantly with the channel depth. These experimental observations are well captured by the depth-averaged model as demonstrated in Fig. 3(b). Moreover, in the shallowest channel with a 32 μm depth, the numerical threshold electric field (326.0 V/cm) closely match the experimental value (305.6 V/cm) with only a 6.6% deviation. The discrepancy, however, increases in a deeper channel because the accuracy of the depth-averaged numerical model is based on the smallness of the channel’s depth-to-width ratio (see the Supplementary Information)^51^.

**Figure 4:**
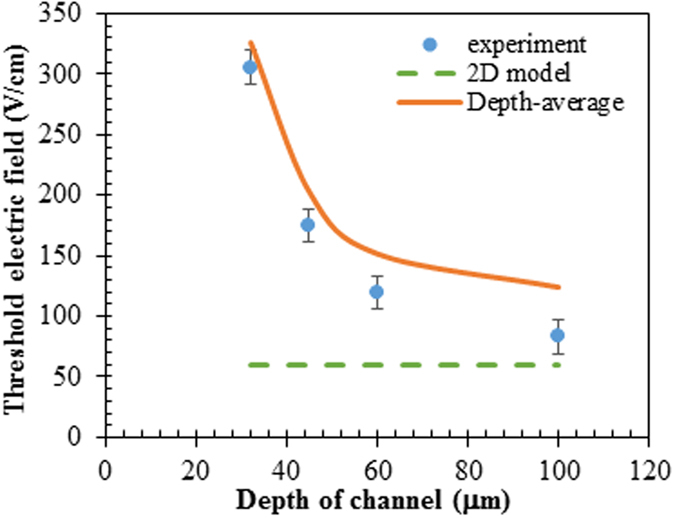
Comparison of the experimental (symbols with error bars) and numerical (solid line for the depth-averaged model and dashed line for the regular 2D model) threshold electric fields for the electrokinetic instability of ferrofluid (0.2× EMG 408) and water co-flow in microchannels of various depths.

Figure 4 compares the experimental (symbols with error bars) and numerical (solid and dashed lines) threshold electric fields in the above tested microchannels. Each experimental data point in the plot is obtained from the averaging of at least three independent measurements, for which the error bar reflects the variation of the applied electric field (calculated from ±25 V divided by the overall 18 mm channel length from the inlet to the outlet reservoir). The numerical data cover those from both the depth-averaged model (solid line) and the regular 2D model (dashed line). As the top and bottom wall effects are completely neglected in the regular 2D model, the numerical threshold electric field remains unvaried at 60.4 V/cm in the four depths of microchannels. This trend is apparently different from the experimental observation. However, the predicted threshold electric field from this model turns out to become comparable to the experimental value for the 100 μm deep channel (83.3 V/cm) with a 27.5% under-prediction. We thus argue that the regular 2D model may be used to estimate the threshold electric field for electrokinetic instability in very deep microchannels (e.g., with a depth-to-width ratio larger than 0.5). In contrast, the depth-averaged model is able to predict the threshold electric field accurately in shallow microchannels (e.g., with a depth-to-width ratio lower than 0.3). It, however, overly considers the stabilizing effects from the top and bottom walls due to the simple averaging in the channel depth direction. Consequently, the threshold electric field is over-predicted in all four depths of microchannels, especially significant in deep ones. Moreover, as Joule heating effects increase with the channel depth^13^,^14^, it becomes important to know how the temperature rise in the co-flowing fluids may affect the interfacial electrokinetic instability and if the influence can be captured by the depth-averaged model. This issue is left for future work.

### Effect of ferrofluid concentration

**Figure 5:**
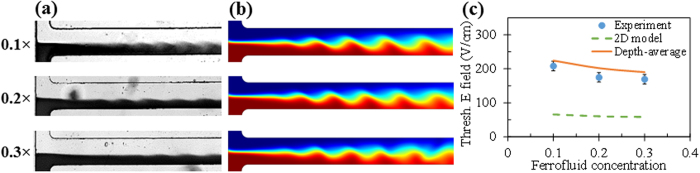
Ferrofluid concentration effect on electrokinetic instability in a 45 μm deep T-shaped microchannel: (**a**) experimental images (dark for ferrofluid and white for water) at the threshold electric fields; (**b**) numerical images (red for ferrofluid and blue for water) at the threshold electric fields; (**c**) comparison of experimental (symbols with error bars) and numerical (solid line for the depth-averaged model and dashed line for the regular 2D model) threshold electric fields.

Figure 5 shows the effect of ferrofluid concentration on the electrokinetic instability in a 45 μm deep microchannel. The instability waves exhibit a visually similar pattern when the ferrofluid concentration is varied from 0.1× to 0.3×. The experimental images at the threshold electric fields in Fig. 5(a) are reasonably simulated by the predicted ferrofluid concentration field from the depth-averaged model in Fig. 5(b). Moreover, as demonstrated in Fig. 5(c), the numerical threshold electric fields are only slightly higher than the experimentally measured values for all three ferrofluid concentrations. The numerical threshold electric fields from the regular 2D model are also included in Fig. 5(c), which, though predicting correctly the decreasing trend of threshold electric field with increasing ferrofluid concentration, are all substantially lower than the experimental values. Moreover, their dependence on ferrofluid concentration is much weaker than the experimental observation. This is again, as explained above, because the top/bottom wall stabilizing effects on electrokinetic flow have been ignored in the regular 2D model.

## Summary

We have extended our earlier work^47^ and developed a depth-averaged model for better understanding and predicting the electrokinetic instability in microchannel ferrofluid/water co-flows. This model considers the top and bottom channel walls’ influences through the depth averaging of the original three-dimensional transport equations. Its validity and accuracy have been tested by comparing the predictions with both the experimental measurements and the predictions of a regular 2D model. We demonstrate that the depth-averaged model is able to capture the experimentally observed dynamic behaviors at the ferrofluid/water interface under different electric fields. It can also predict with a close agreement the measured threshold electric fields for ferrofluids of different concentrations in shallow microchannels. The accuracy of this model is, however, compromised in deep microchannels due to the breakdown of the assumption of small channel depth-to-width ratio in the asymptotic analysis (see the Supplementary Information). For very deep microchannels (e.g., with a channel depth-to-width ratio higher than 0.5), the regular 2D model may be used to estimate the threshold electric field because the top and bottom channel walls’ stabilizing effects on electrokinetic flow become insignificant. We will study in future work the role of Joule heating in electrokinetic instability of co-flowing fluids with conductivity mismatch.

## Experiment

**Figure 6:**
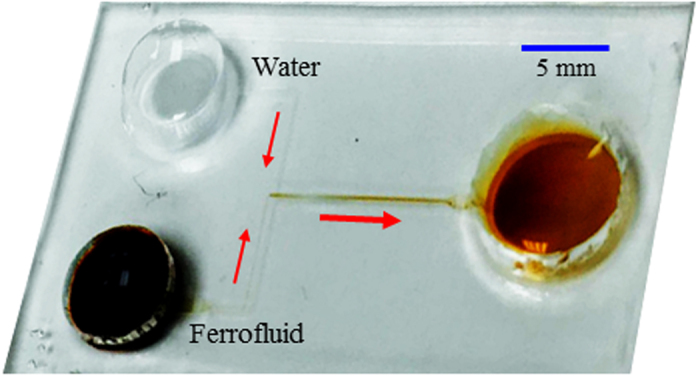
Picture of the T-shaped microchannel used in experiments where the block arrows indicate the flow directions. The two inlet reservoirs are, respectively, filled with ferrofluid (dark) and water (transparent) that are mixed in the main-branch by electrokinetic instability and collected into the outlet reservoir (brown).

Figure 6 shows a picture of the T-shaped microchannel that was fabricated using polydimethylsiloxane (PDMS) with the standard soft lithography technique. The detailed fabrication procedure can be referred to our earlier work^47^. The two side-branches of the microchannel are each 8 mm long with a width of 100 μm, and the main-branch is 10 mm long with a 200 μm width. Four depths of channels were fabricated in order to test the validity and accuracy of the developed depth-averaged numerical model. The channel depth was measured under a microscope, which was found to be 32 μm, 45 μm, 60 μm and 100 μm, respectively. Ferrofluid was prepared at three different concentrations, 0.1×, 0.2× and 0.3× by volume fraction, of EMG 408 ferrofluid (Ferrotec). The original ferrofluid is a stable colloidal suspension of 1.2% (volume ratio) magnetic nanoparticles (made of magnetite, Fe_3_O_4_) with an average diameter of 10 nm in pure water (Ferrotec).

Prior to experiment, all reservoirs were emptied. Equal volume of DI water and ferrofluid were dispensed into the two inlet reservoirs of the microchannel. Immediately following that, DI water of the same volume was added to the outlet reservoir to match the liquid level in the inlet reservoirs for pressure balances. Electric field was generated by imposing an equal magnitude of DC voltage (Glassman High Voltage Inc.) to the two inlet reservoirs. The outlet reservoir was grounded. The flow instability at the ferrofluid and water interface was visualized at the T-junction of the microchannel using an inverted microscope (Nikon Eclipse TE2000U, Nikon Instruments). Digital videos were recorded through a CCD camera (Nikon DS-Qi1Mc) at a rate of around 15 frames per second. The obtained images were post-processed using the Nikon imaging software (NIS-Elements AR 2.30).

## Simulation

**Figure 7:**
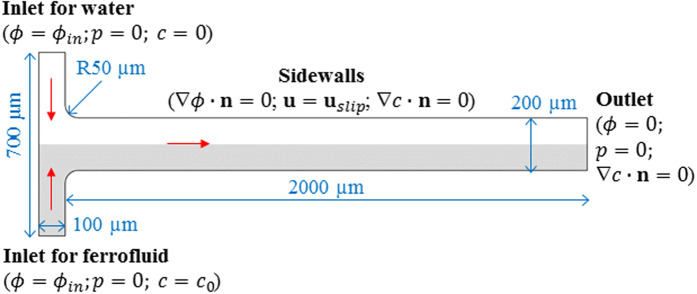
Computational domain for the depth-averaged numerical model, where the gray area represents ferrofluid and the white area represents water at the initial state. Boundary conditions are presented for the charge, fluid and mass transfer equations in order. The definitions of the symbols are referred to the text.

A nonlinear depth-averaged numerical model was developed to simulate the electrokinetic instability in microchannel ferrofluid and water co-flows. It solved the depth-averaged governing equations in the horizontal plane of the microchannel. These equations, as presented below, were obtained from a second-order asymptotic analysis (refer to Lin *et al*.^51^ for the approach) in shallow microchannels by defining the channel depth-to-width ratio as a smallness parameter. The detailed process for this asymptotic analysis is given in the Supplementary Information due to space limit. Figure 7 shows the computational domain used in our depth-averaged numerical model, which considers a reduced length for both the main-branch and side-branches to save computational time. The specific channel dimensions are labeled in Fig. 7.

### Governing equations and boundary conditions

#### Electric field

The depth-averaged governing equation for electric field is given by^51^,^52^


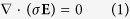


where *σ* is the electric conductivity, and 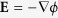 is the depth-averaged electric field in the horizontal plane of the microchannel with *ϕ* being the electric potential. As highlighted in the computational domain, an equal electric potential, *ϕ* = *ϕ*_*in*_, is applied to the two inlets while *ϕ* = 0 (i.e., grounded) is applied to the outlet. The electric field is assumed to be confined within the fluid, and thus an electrically insulating condition is imposed upon the channel sidewalls, i.e., 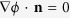 with **n** denoting the unit normal vector.

#### Flow field

The depth-averaged continuity and momentum equations for flow field are giving by^51^,^52^


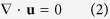



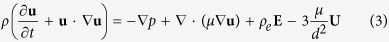



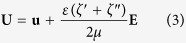


where **u** is the depth-averaged fluid velocity in the horizontal plane of the microchannel, *ρ* is the fluid density, *t* is the time, *p* is the pressure, *μ* is the fluid viscosity, 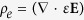 is the free charge density from Poisson’s equation^2^,^3^ with *ε* being the fluid permittivity, *d* is the half-depth (in the *z*-direction) of the microchannel, *ζ*′ and *ζ*″ are the zeta potentials of the top and bottom channel walls, respectively. The third term on the right hand side of Eq. (3) represents the Coulomb force^15^. The last term results from the depth-averaging analysis, which accounts for the influences of the top and bottom channel walls on the flow field as viewed from the definite of **U** in Eq. (4). Under the condition of thin electric double layer that is fulfilled in our experiments^47^, a Helmholtz–Smoluchowski electroosmotic slip velocity, **u**_*slip*_, is imposed upon the channel sidewalls^2^,^3^


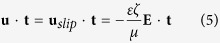


where **t** is the unit tangential vector, and *ζ* is the zeta potential of the channel sidewalls. At the inlet and outlet, an equal pressure, *p* = 0, is applied. The entire fluid of ferrofluid and water is assumed stationary at the initial state.

#### Concentration field

The depth-averaged convection-diffusion equation for the transfer of ferrofluid nanoparticles is given by^51^


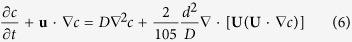


where *c* is the concentration of ferrofluid nanoparticles (or simply speaking, ferrofluid concentration), *D* is the diffusivity of ferrofluid nanoparticles, and **U** is defined in Eq. (4). Similarly, the last term in Eq. (6) comes from the depth-averaging analysis, which reflects the effect of the depth-averaging flow field on mass transfer. To solve Eq. (6), *c* = 0 and *c* = *c*_0_ are applied to the water and ferrofluid inlets, respectively, with *c*_0_ being the ferrofluid concentration in the inlet reservoir. The channel sidewalls are assumed non-penetrating to ferrofluid nanoparticles, i.e., 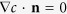. A similar condition is also imposed to the outlet, which represents a fully developed concentration field. At the initial state, the concentration is assumed to be uniformly 0 and *c*_0_ in the water and ferrofluid halves of the computation domain, respectively.

### Numerical method

The above depth-averaged governing equations for electric, flow and concentration fields are coupled through the concentration dependence of fluid electric conductivity, *σ*, and viscosity, *μ*. We used the same formulae as those in earlier studies^47^,^53^,^54^,^55^,^56^


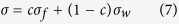



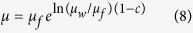


where the subscripts *f* and *w* denote the properties of the original ferrofluid and water, respectively. Note that the fluid density variation with ferrofluid concentration is small (less than 3%) and has been neglected in this work. Also ignored is the variation of fluid permittivity with ferrofluid concentration because its influence on electrokinetic flow instability has been estimated to be much smaller than that of electric conductivity^57^. The average (or effective) zeta potential of the channel walls was measured to be around −0.075 V (or 75 mV) via the electric current monitoring method^58^. This value was found to be a weak function of ferrofluid concentration due probably to the very low volume ratio of the suspended magnetic nanoparticles (0.12%, 0.24% and 0.36% for 0.1×, 0.2× and 0.3× EMG 408 ferrofluids, respectively). We therefore used the same zeta potential for the ferrofluid and the water flows in our model. However, considering that the zeta potential of glass is typically smaller than that of PDMS^59^, we used −0.08 V for the top and side PDMS walls and −0.06 V for the bottom glass wall in the model. Other parameters involved in the simulation are similar to those in the 2D regular model in our previous paper^47^ and listed in Table 1.

The 2D depth-averaged numerical model was developed in finite element-based commercial software, COMSOL^®^ 5.2. The governing equations for charge, fluid and mass transport were solved using the “Laminar Flow”, “Electric Currents” and “Transport of Diluted Species” modules, respectively. The additional terms that result from the depth-averaging analysis in Eqs (3) and (6) were added to the model via the “Force” feature in the “Laminar Flow” module and the “Reaction” feature in the “Transport of Diluted Species” module, respectively. A similar mesh setting to the regular 2D model (i.e., the additional depth-averaged terms in Eqs (3) and (6) are dropped out) in our previous paper^47^ was used in this work. It was a structured mesh with 4 μm-sized square elements in all the branches and even smaller triangular elements in the fillet regions. More details on the mesh and grid independence study are referred to ref. 47.

## Additional Information

**How to cite this article:** Song, L. *et al*. Electrokinetic instability in microchannel ferrofluid/water co-flows. *Sci. Rep.* **7**, 46510; doi: 10.1038/srep46510 (2017).

**Publisher's note:** Springer Nature remains neutral with regard to jurisdictional claims in published maps and institutional affiliations.

## Supplementary Material

Supplementary Information

## Figures and Tables

**Table 1:** Material properties of the ferrofluid-water-microchannel system used in the depth-averaged and 2D regular models.

Symbol	Description	Value
*ρ*	Density of fluid	1000 kg/m^3^
*μ*_*w*_	Viscosity of water	1e-3 Pa · S
*μ*_*f*_	Viscosity of 1× ferrofluid	2e-3 Pa · S
*ε*	Fluid permittivity	7.083e-10 C^2^/J · m
*σ*_*w*_	Electric conductivity of water	10e-4 S/m
*σ*_*f*_	Electric conductivity of 1× ferrofluid	5314.5e-4 S/m
*ζ*	Zeta potential of PDMS sidewalls	−0.08 V
*ζ*′	Zeta potential of top PDMS wall	−0.08 V
*ζ*″	Zeta potential of bottom glass wall	−0.06 V
*D*	Diffusion coefficient of ferrofluid	1e-9 m^2^/s
