# Supplementary material for: Electrokinetic instability in microchannel ferrofluid/water co-flows
Source: Sci Rep. 2017 Apr 13;7:46510. doi: 10.1038/srep46510 (PMC5390253; doi:10.1038/srep46510)
Supplement: Supplementary Information [file srep46510-s1.pdf]

Supplementary Information for:

# Electrokinetic instability in microchannel ferrofluid/water co-flows

Le Song,[1] Liandong Yu,[1,*] Yilong Zhou,[2] Asher Reginald Antao,[2] Rama Aravind Prabhakaran,[2] Xiangchun Xuan[2,*]

[1] School of Instrument Science and Opto-electronic Engineering, Hefei University of Technology, Hefei 230009, China

[2] Department of Mechanical Engineering, Clemson University, Clemson, SC 29634-0921, USA

[*]Corresponding authors. Emails: xcxuan@clemson.edu (Dr. Xuan) and liandongyu@hfut.edu.cn (Dr. Yu).

**Depth-Averaged Asymptotic Analysis**

Under the assumptions of rapid charge relaxation, electro-neutrality, and a thin electrical double layer, we have

$$\nabla \cdot (\sigma \mathbf{E}) = 0 \tag{S1}$$

$$\nabla \cdot (\epsilon \mathbf{E}) = \rho_e \tag{S2}$$

$$\nabla \cdot \boldsymbol{v} = 0 \tag{S3}$$

$$\rho \left( \frac{\partial \boldsymbol{v}}{\partial t} + \boldsymbol{v} \cdot \nabla \boldsymbol{v} \right) = -\nabla p + \nabla \cdot (\mu \nabla \boldsymbol{v}) + \rho_e \mathbf{E} \tag{S4}$$

$$\frac{\partial c}{\partial t} + \boldsymbol{v} \cdot \nabla c = D \nabla^2 c \tag{S5}$$

Where $\rho_e$ is the charge density, $\boldsymbol{v} = \boldsymbol{v}(u, v, w)$ is the three-dimensional velocity vector. Eq. (S1) and (S2) are used to calculate the electric field, Eq. (S3) and (S4) are used to study the flow field, and the Eq. (S5) based on the species conservation is used for observing the dynamic changing process of concentration.

The boundary conditions at the channel wall are listed below,

$$\nabla \phi \cdot \mathbf{n} = 0 \tag{S6}$$

$$\nabla c \cdot \mathbf{n} = 0 \tag{S7}$$

$$\boldsymbol{v} \cdot \mathbf{t} = -\frac{\epsilon \zeta \mathbf{E} \cdot \mathbf{t}}{\mu} \tag{S8}$$

$$\boldsymbol{v} \cdot \mathbf{n} = 0 \tag{S9}$$

To non-dimensionalize our governing equations, following scales which are similar with those in Lin et. al. [1] are used here

$[x, y] = H$; $[z] = d$; $[\sigma] = \sigma_0$; $[c] = c_0$; $[\mu] = \mu_0$; $[\phi] = \phi_0 = E_0 H$; $[u, v] = U_{ev} \equiv \frac{\epsilon E_0^2 d^2}{\mu_0 H}$; $[w] = \frac{U_{ev} d}{H}$;

$[t] = t_0 = \frac{H}{U_{ev}}$; $[p] = \frac{\mu_0 U_{ev} H}{d^2}$;

Where $H$ and $d$ denote the half-width (in the y-direction) and half-depth (in the z-direction) of channel, respectively, $\sigma_0$, $c_0$, $\mu_0$ and $\zeta_0$ are the reference electric conductivity, concentration, viscosity and zeta potential of fluid, the characteristic electric field $E_0$ is taken to be the applied electric field in the channel, and $U_{ev}$ is the electroviscous velocity.

After the non-dimensionalization, the governing equations then be written as

$$\delta^2\nabla_H\cdot(\sigma\nabla_H\phi)+\frac{\partial}{\partial z}\left(\sigma\frac{\partial\phi}{\partial z}\right)=0 \tag{S10}$$

$$\nabla_H\cdot\mathbf{u}+\frac{\partial w}{\partial z}=0 \tag{S11}$$

$$R_{ed}\delta\left(\frac{\partial\mathbf{u}}{\partial t}+\boldsymbol{v}\cdot\nabla\mathbf{u}\right)=-\nabla_H p+\delta^2\nabla_H\cdot(\mu\nabla_H\mathbf{u})+\frac{\partial}{\partial z}\left(\mu\frac{\partial\mathbf{u}}{\partial z}\right)+\left(\nabla_H^2\phi+\frac{1}{\delta^2}\frac{\partial^2\phi}{\partial z^2}\right)\nabla_H\phi \tag{S12}$$

$$R_{ed}\delta^3\left(\frac{\partial w}{\partial t}+\boldsymbol{v}\cdot\nabla w\right)=-\frac{\partial p}{\partial z}+\delta^4\nabla_H\cdot(\mu\nabla_H w)+\delta^2\frac{\partial}{\partial z}\left(\mu\frac{\partial w}{\partial z}\right)+\left(\nabla_H^2\phi+\frac{1}{\delta^2}\frac{\partial^2\phi}{\partial z^2}\right)\frac{\partial\phi}{\partial z} \tag{S13}$$

$$P_{ed}\delta\left(\frac{\partial c}{\partial t}+\boldsymbol{v}\cdot\nabla c\right)=\delta^2\nabla_H^2 c+\frac{\partial^2 c}{\partial z^2} \tag{S14}$$

Where $\delta\equiv d/H$ is a smallness parameter, $\mathbf{u}=\mathbf{u}(u,v)$ is two-dimensional velocity vector, $P_{ed}\equiv U_{ev}d/D$ and $R_{ed}\equiv\rho U_{ev}d/\mu_0$ are the Peclet and Reynolds numbers, respectively, and $\nabla_H$ is the two-dimensional (x, y) differential operator defined below,

$$\nabla_H\equiv\left(\frac{\partial}{\partial x},\frac{\partial}{\partial y}\right) \tag{S15}$$

Except for the electroosmotic velocity, boundary conditions keep the same form after the non-dimensionalization. Considering the dissimilar boundary conditions of top and bottom wall, we have,

$$\mathbf{u}'_{eo}=\frac{\zeta' H}{\mu E_0 d^2}\nabla\phi \tag{S16}$$

$$\mathbf{u}''_{eo}=\frac{\zeta'' H}{\mu E_0 d^2}\nabla\phi \tag{S17}$$

Here $\zeta'$ is the zeta potential of top wall, $\zeta''$ is the zeta potential of bottom wall, $\mathbf{u}'_{eo}$ and $\mathbf{u}''_{eo}$ are the electroosmotic velocity at top and bottom wall, respectively.

Under the assumption of $\delta\ll 1$, the asymptotic analysis introduced in Lin et al. [1] is performed here for our study. The depth-averaged functions are defined as

$$\bar{f}=\frac{1}{2}\int_{-1}^{1}f\,dz \tag{S18}$$

$$f=f_0+\delta f_1+\delta^2 f_2+\ldots \tag{S19}$$

Where the subscript stands for the corresponding order variables. In this paper, we perform the depth-averaged analysis up to the second order in $\delta$.

**1. Electric field equation**

Performing $\delta^0$ order balance for Eq. (S10), we get

$$\frac{\partial}{\partial z}\left(\sigma_0 \frac{\partial \phi_0}{\partial z}\right) = 0 \tag{S20}$$

Considering $\sigma_0 = \sigma_0(x, y, t)$ only, we obtain

$$\frac{\partial}{\partial z}\left(\frac{\partial \phi_0}{\partial z}\right) = 0 \tag{S21}$$

Applying the insulating boundary conditions $\frac{\partial \phi_0}{\partial z} = 0$, at $z = \pm 1$,

$$\frac{\partial \phi_0}{\partial z} = 0,\ \phi_0 = \phi_0(x, y, t) \tag{S22}$$

Performing a $\delta^1$ order on Eq. (S10) balance and performing the same steps as before, we get

$$\left(\frac{\partial \phi_1}{\partial z}\right) = 0 \tag{S23}$$

Similarly for a $\delta^2$order balance:

$$\nabla_H \cdot (\sigma_0 \nabla_H \phi_0) + \frac{\partial}{\partial z}\left(\sigma_0 \frac{\partial \phi_2}{\partial z}\right) = 0 \tag{S24}$$

Applying the insulating boundary conditions at the top and bottom surfaces for $\phi_2$ and simplifying,

$$\nabla_H \cdot (\sigma_0 \nabla_H \phi_0) = 0 \tag{S25}$$

From Eq. (S24) and (S25), we obtain,

$$\left(\frac{\partial \phi_2}{\partial z}\right) = 0 \tag{S26}$$

For $\delta^3$order balance of Eq. (S10):

$$\nabla_H \cdot (\sigma_1 \nabla_H \phi_0) + \nabla_H \cdot (\sigma_0 \nabla_H \phi_1) + \frac{\partial}{\partial z}\left(\sigma_0 \frac{\partial \phi_3}{\partial z}\right) = 0 \tag{S27}$$

Applying boundary conditions for $\phi_3$,

$$\nabla_H \cdot (\sigma_1 \nabla_H \phi_0) + \nabla_H \cdot (\sigma_0 \nabla_H \phi_1) = 0 \tag{S28}$$

Performing (S25) $+\delta\times$(S28), we obtain the final depth-averaged equation for electric field,

$$\nabla_H \cdot (\bar{\sigma}\, \nabla_H \bar{\phi}) = 0 \tag{S29}$$

## 2. Flow equation

Performing a $\delta^0$ order balance for Eq. (S11) – (S13):

$$\nabla_H \cdot \mathbf{u}_0 + \frac{\partial w_0}{\partial z} = 0 \tag{S30}$$

$$0 = -\nabla_H p_0 + \frac{\partial}{\partial z}\left(\mu \frac{\partial \mathbf{u}_0}{\partial z}\right) + \nabla_H^2 \phi_0 \nabla_H \phi_0 \tag{S31}$$

$$0 = -\frac{\partial p_0}{\partial z} \tag{S32}$$

In this paper, we consider $\mu = \mu(x, y, t)$ only. From Eq. (S32), we get

$$p_0 = p_0(x, y, t) \tag{S33}$$

Integrating Eq. (S31), yielding

$$\mathbf{u}_0 = \left(\frac{\nabla_H p_0 - \nabla_H^2 \phi_0 \nabla_H \phi_0}{\mu}\right)\frac{1}{2} z^2 + A_0 z + A_1 \tag{S34}$$

Where $A_0$ and $A_1$ are constants of integration.

Applying the boundary condition $\mathbf{u}_0 = \mathbf{u}'_{eo0}$ at $z = 1$, and $\mathbf{u}_0 = \mathbf{u}''_{eo0}$ at $z = -1$, we get

$$\mathbf{u}_0 = \mathbf{U}_0 + \frac{1}{2}(\mathbf{u}'_{eo0} - \mathbf{u}''_{eo0})z + \frac{1}{2}(\mathbf{u}'_{eo0} + \mathbf{u}''_{eo0}) \tag{S35}$$

Where

$$\mathbf{U}_0 = \left(\frac{\nabla_H p_0 - \nabla_H^2 \phi_0 \nabla_H \phi_0}{\mu}\right)\left(\frac{z^2 - 1}{2}\right) \tag{S36}$$

Upon depth-averaging, we get,

$$\overline{\mathbf{u}_0} = -\frac{1}{3}\left(\frac{\nabla_H p_0 - \nabla_H^2 \phi_0 \nabla_H \phi_0}{\mu}\right) + \frac{1}{2}(\mathbf{u}'_{eo0} + \mathbf{u}''_{eo0}) \tag{S37}$$

$$\overline{\mathbf{U}_0} = -\frac{1}{3}\left(\frac{\nabla_H p_0 - \nabla_H^2 \phi_0 \nabla_H \phi_0}{\mu}\right) \tag{S38}$$

So,

$$\mathbf{u}_0 = \overline{\mathbf{u}_0} + \overline{\mathbf{U}_0}\left(\frac{1}{2} - \frac{3}{2} z^2\right) + \frac{1}{2}(\mathbf{u}'_{eo0} - \mathbf{u}''_{eo0})z \tag{S39}$$

Depth-averaging Eq. (S30) and applying the boundary condition $w_0 = 0$ at $z = \pm 1$, we get

$$\nabla_H \cdot \overline{\mathbf{u}_0} + \frac{1}{2}\int_{-1}^{1} \frac{\partial w_0}{\partial z} dz = 0 \tag{S40}$$

So we have

$$\nabla_H \cdot \overline{\mathbf{u}_0} = 0 \tag{S41}$$

Integrating Eq. (S30), we get

$$w_0 = -\int \nabla_H \cdot \mathbf{u}_0 dz = \nabla_H \cdot \overline{\mathbf{U}_0}\left(\frac{1}{2}z^3 - \frac{1}{2}z\right) + A_2 \tag{S42}$$

Where $A_2$ is constant of integration.

Applying the boundary condition $w_0 = 0$ at z $= \pm 1$, we get

$$w_0 = \nabla_H \cdot \overline{\mathbf{U}_0}\left(\frac{1}{2}z^3 - \frac{1}{2}z\right) \tag{S43}$$

The $\delta^1$ order balance for Eq. (S11) – (S13) gives

$$\nabla_H \cdot \mathbf{u}_1 + \frac{\partial w_1}{\partial z} = 0 \tag{S44}$$

$$R_{ed}\left(\frac{\partial \mathbf{u}_0}{\partial t} + \mathbf{u}_0 \cdot \nabla_H \mathbf{u}_0 + w_0 \frac{\partial \mathbf{u}_0}{\partial z}\right) = -\nabla_H p_1 + \frac{\partial}{\partial z}\left(\mu \frac{\partial \mathbf{u}_1}{\partial z}\right) + \nabla_H^2 \phi_0 \nabla_H \phi_1 + \nabla_H^2 \phi_1 \nabla_H \phi_0 + \frac{\partial^2 \phi_3}{\partial z^2}\nabla_H \phi_0 \tag{S45}$$

$$0 = -\frac{\partial p_1}{\partial z} \tag{S46}$$

From Eq. (S46) we have $p_1 = p_1(x, y, t)$.

Depth-averaging Eq. (S44) and applying the boundary condition, we can get,

$$\nabla_H \cdot \overline{\mathbf{u}_1} = 0 \tag{S47}$$

Integrating Eq. (S27), we get

$$\sigma_0 \phi_3 = \frac{1}{48} P_{ed}[\nabla_H(\overline{U_0} \cdot \nabla_H \sigma_0)\nabla_H \phi_0]\left(\frac{7}{5}z^2 - z^4 + \frac{1}{5}z^6\right) \tag{S48}$$

Rearranging the Eq. (S48) we obtain

$$\phi_3 = \frac{1}{48} P_{ed}[\nabla_H(\overline{U_0} \cdot \nabla_H ln\sigma_0) \cdot \nabla_H \phi_0]\left(\frac{7}{5}z^2 - z^4 + \frac{1}{5}z^6\right) \tag{S49}$$

Integrating Eq. (S45) and performing the depth-averaging actions, we can get

$$\overline{\mathbf{u}_1} = \frac{R_{ed}}{\mu}\left[-\frac{1}{3}\left(\frac{\partial\overline{\mathbf{u}_0}}{\partial t} + \overline{\mathbf{u}_0}\cdot\nabla_H\overline{\mathbf{u}_0}\right) - \frac{1}{15}\left(\frac{\partial\overline{\mathbf{U}_0}}{\partial t} + \overline{\mathbf{u}_0}\cdot\nabla_H\overline{\mathbf{U}_0} + \overline{\mathbf{U}_0}\cdot\nabla_H\overline{\mathbf{u}_0}\right)\right.$$
$$\left. - \frac{1}{21}(\overline{\mathbf{U_0}}\cdot\nabla_H\overline{\mathbf{U_0}}) - \frac{2}{35}\overline{\mathbf{U}_0}(\nabla_H\cdot\overline{\mathbf{U}_0})\right]$$
$$- \frac{1}{3}\left(\frac{\nabla_H p_1 - \nabla_H^2\phi_0\nabla_H\phi_1 - \nabla_H^2\phi_1\nabla_H\phi_0}{\mu}\right) \tag{S50}$$
$$+ \frac{2}{315}\frac{P_{ed}[\nabla_H(\overline{\mathbf{U}_0}\cdot\nabla_H ln\sigma_0)\cdot\nabla_H\phi_0]\nabla_H\phi_0}{\mu} + \frac{1}{2}(\mathbf{u}'_{eo1} + \mathbf{u}''_{eo1})$$

Neglecting the higher order terms,

$$\overline{\mathbf{u}_1} = \frac{R_{ed}}{\mu}\left[-\frac{1}{3}\left(\frac{\partial\overline{\mathbf{u}_0}}{\partial t} + \overline{\mathbf{u}_0}\cdot\nabla_H\overline{\mathbf{u}_0}\right)\right] - \frac{1}{3}\left(\frac{\nabla_H p_1 - \nabla_H^2\phi_0\nabla_H\phi_1 - \nabla_H^2\phi_1\nabla_H\phi_0}{\mu}\right)$$
$$+ \frac{1}{2}(\mathbf{u}'_{eo1} + \mathbf{u}''_{eo1}) \tag{S51}$$

$$\overline{\mathbf{U}_1} = \frac{R_{ed}}{\mu}\left[-\frac{1}{3}\left(\frac{\partial\overline{\mathbf{u}_0}}{\partial t} + \overline{\mathbf{u}_0}\cdot\nabla_H\overline{\mathbf{u}_0}\right)\right] - \frac{1}{3}\left(\frac{\nabla_H p_1 - \nabla_H^2\phi_0\nabla_H\phi_1 - \nabla_H^2\phi_1\nabla_H\phi_0}{\mu}\right) \tag{S52}$$

On rearranging,

$$Re_d\left(\frac{\partial\overline{\mathbf{u}_0}}{\partial t} + \overline{\mathbf{u}_0}\cdot\nabla_H\overline{\mathbf{u}_0}\right)$$
$$= -\nabla_H p_1 + \nabla_H^2\phi_1\nabla_H\phi_0 + \nabla_H^2\phi_0\nabla_H\phi_1 - 3\mu\left(\overline{\mathbf{u}_1} - \frac{\mathbf{u}'_{eo1} + \mathbf{u}''_{eo1}}{2}\right) \tag{S53}$$

Similarly for $\delta^2$ balance, we obtain

$$Re_d\left(\frac{\partial\overline{\mathbf{u}_1}}{\partial t} + \overline{\mathbf{u}_1}\cdot\boldsymbol{\nabla}_H\overline{\mathbf{u}_0} + \overline{\mathbf{u}_0}\cdot\nabla_H\overline{\mathbf{u}_1}\right)$$
$$= -\nabla_H\overline{p_2} + \nabla_H^2\phi_0\nabla_H\phi_2 + \nabla_H^2\phi_2\nabla_H\phi_0 + \nabla_H^2\phi_1\nabla_H\phi_1 \tag{S54}$$
$$- 3\mu\left(\overline{\mathbf{u}_2} - \frac{\mathbf{u}'_{eo2} + \mathbf{u}''_{eo2}}{2}\right) + \nabla_H\cdot(\mu\nabla_H\overline{\mathbf{u}_0})$$

$$\nabla_H\cdot\overline{\mathbf{u}_2} = 0 \tag{S55}$$

By (S37) + $\delta$ × (S53) + $\delta^2$ × (S54) we obtain,

$$Re_d\delta\left(\frac{\partial\overline{\mathbf{u}}}{\partial t} + \overline{\mathbf{u}}\cdot\nabla_H\overline{\mathbf{u}}\right)$$
$$= -\nabla_H\bar{p} + \delta^2\nabla_H\cdot(\mu\nabla_H\overline{\mathbf{u}}) + \nabla_H^2\bar{\phi}\nabla_H\bar{\phi} - 3\mu\left(\overline{\mathbf{u}} - \frac{\mathbf{u}'_{eo} + \mathbf{u}''_{eo}}{2}\right) \tag{S56}$$

Combining Eq. (S41), (S47) and (S54), we have

$$\nabla_H \cdot \overline{\mathbf{u}} = 0 \tag{S57}$$

**3. Concentration equation**

At the $\delta^0$-order, we have

$$\frac{\partial^2 c_0}{\partial z^2} = 0 \tag{S58}$$

Applying the boundary condition $\frac{\partial c_0}{\partial z} = 0$ at $z = \pm 1$

$$c_0 = c_0(x, y, t) \tag{S59}$$

At the $\delta^1$-order, we have

$$P_{ed}\left(\frac{\partial c_0}{\partial t} + \mathbf{u}_0 \cdot \nabla_H c_0\right) = \frac{\partial^2 c_1}{\partial z^2} \tag{S60}$$

Depth-averaging and applying the boundary condition,

$$\int_{-1}^{1} \frac{\partial^2 c_1}{\partial z^2} dz = \int_{-1}^{1} P_{ed}\left(\frac{\partial c_0}{\partial t} + \mathbf{u}_0 \cdot \nabla_H c_0\right) dz \tag{S61}$$

So, we have

$$\frac{\partial c_0}{\partial t} + \overline{\mathbf{u}_0} \cdot \nabla_H c_0 = 0 \tag{S62}$$

Eq. (S60) can also be integrated in the z-direction,

$$\frac{\partial c_1}{\partial z} = P_{ed}\left[\frac{\partial c_0}{\partial t} z + (\overline{\mathbf{u}_0} \cdot \nabla_H c_0) z + (\overline{\mathbf{U}}_0 \cdot \nabla_H c_0)\left(\frac{1}{2} z - \frac{1}{2} z^3\right)\right] + A_3 \tag{S63}$$

Where $A_3$ is constant of integration.

Based on Eq. (S62) and applying the boundary condition, we obtain

$$\frac{\partial c_1}{\partial z} = P_{ed}(\overline{\mathbf{U}_0} \cdot \nabla_H c_0)\left(\frac{1}{2} z - \frac{1}{2} z^3\right) \tag{S64}$$

Integrating Eq. (S64) and applying the depth-averaging action, we get

$$c_1 = \overline{c_1} + \frac{1}{4} P_{ed}(\overline{\mathbf{U}_0} \cdot \nabla_H c_0)\left(-\frac{7}{30} + z^2 - \frac{1}{2} z^4\right) \tag{S65}$$

At the $\delta^2$-order, we have

$$P_{ed}\left(\frac{\partial c_1}{\partial t} + \mathbf{u}_1 \cdot \nabla_H c_0 + \mathbf{u}_0 \cdot \nabla_H c_1 + w_0 \frac{\partial c_1}{\partial z}\right) = \nabla_H^2 c_0 + \frac{\partial^2 c_2}{\partial z^2} \tag{S66}$$

Depth-averaging and applying the boundary condition, we get

$$P_{ed}\left(\frac{\partial\overline{c_1}}{\partial t}+\overline{\mathbf{u}_1}\cdot\nabla_H c_0+\overline{\mathbf{u}_0\cdot\nabla_H c_1}+\overline{w_0\frac{\partial c_1}{\partial z}}\right)=\nabla_H^2 c_0 \tag{S67}$$

Using the expressions for $\mathbf{u}_0$, $w_0$ and $c_1$, Eq. (S67) can be expanded as

$$\begin{aligned}\frac{\partial\overline{c_1}}{\partial t}&+\overline{\mathbf{u}_1}\cdot\nabla_H c_0+\overline{\mathbf{u}_0}\cdot\nabla_H\overline{c_1}\\ &=\frac{1}{P_{ed}}\nabla_H^2 c_0\\ &+\frac{2}{105}P_{ed}[(\overline{\mathbf{U}_0}\cdot\nabla_H)(\overline{\mathbf{U}_0}\cdot\nabla_H c_0)+(\nabla_H\cdot\overline{\mathbf{U}_0})(\overline{\mathbf{U}_0}\cdot\nabla_H c_0)]\end{aligned} \tag{S68}$$

Performing (S62) + $\delta$ ×(S68), we obtain

$$\frac{\partial\bar{c}}{\partial t}+\bar{\mathbf{u}}\cdot\nabla_H\bar{c}=\frac{\delta}{P_{ed}}\left\{\nabla_H^2\bar{c}+\frac{2}{105}P_{ed}^2[(\bar{\mathbf{U}}\cdot\nabla_H)(\bar{\mathbf{U}}\cdot\nabla_H\bar{c})+(\nabla_H\cdot\bar{\mathbf{U}})(\bar{\mathbf{U}}\cdot\nabla_H\bar{c})]\right\} \tag{S69}$$

Where

$$\bar{\mathbf{U}}=\bar{\mathbf{u}}-\frac{\mathbf{u}'_{eo}+\mathbf{u}''_{eo}}{2} \tag{S70}$$

## 4. Summary

Upon the dimensionless nonlinear depth-averaged equations (S29), (S55), (S56) and (S68), we now transform the equations to dimensional form

$$\frac{\partial c}{\partial t}+\mathbf{u}\cdot\nabla c=D\nabla^2 c+\frac{2}{105}\frac{d^2}{D}[(\mathbf{U}\cdot\nabla)(\mathbf{U}\cdot\nabla c)+(\nabla\cdot\mathbf{U})(\mathbf{U}\cdot\nabla c)] \tag{S71}$$

$$\nabla\cdot(\sigma\,\nabla\phi)=0 \tag{S72}$$

$$\nabla\cdot\mathbf{u}=0 \tag{S73}$$

$$\rho\left(\frac{\partial\mathbf{u}}{\partial t}+\mathbf{u}\cdot\nabla\mathbf{u}\right)=-\nabla p+\epsilon\nabla^2\phi\nabla\phi+\nabla\cdot(\mu\nabla\mathbf{u})-3\frac{\mu}{d^2}\mathbf{U} \tag{S74}$$

Where

$$\mathbf{U}=\mathbf{u}-\frac{\epsilon(\zeta'+\zeta'')}{2\mu}\nabla\phi \tag{S75}$$

## References


1. H. Lin, B. D. Storey and J. G. Santiago, J. Fluid Mech. 608, 43-70 (2008).
